# The Placebo and Nocebo Responses in Clinical Trials in Inflammatory Bowel Diseases

**DOI:** 10.3389/fphar.2021.641436

**Published:** 2021-03-31

**Authors:** Paul Enck, Sibylle Klosterhalfen

**Affiliations:** Department of Internal Medicine VI: Psychosomatic Medicine and Psychotherapy, University Hospital Tübingen, Tübingen, Germany

**Keywords:** placebo and nocebo effects, clinical trial, systematic (literature) review, inflammatory bowel disease, Crohn´s disease, ulcerative colitis

## Abstract

Placebo and nocebo responses are mostly discussed in clinical trials with functional bowel disorders. Much less has been investigated and is known in gastrointestinal diseases beyond irritable bowel syndrome (IBS), especially in inflammatory bowel diseases (IBD). For the purpose of this review, we screened the Journal of Interdisciplinary Placebo Studies (JIPS) database with approximately 4,500 genuine placebo research articles and identified nine meta-analyses covering more than 135 randomized and placebo-controlled trials (RCTs) with more than 10,000 patients with Crohn´s disease (CD) and another five meta-analyses with 150 RCTs and more than 10,000 patients with ulcerative colitis (UC). Only three discussed nocebo effects, especially in the context of clinical use of biosimilars to treat inflammation. The articles were critically analyzed with respect to the size of the placebo response in CD and UC, its effects on clinical improvement versus maintenance of remission, and mediators and moderators of the response identified. Finally, we discussed and compared the differences and similarities of the placebo responses in IBD and IBS and the nocebo effect in switching from biologics to biosimilars in IBD management.

## Introduction

According to consented definitions, “placebo and nocebo response includes all health changes that result after administration of an inactive treatment (i.e., differences in symptoms before and after treatment), thus including natural history and regression to the mean. The placebo and nocebo effect refers to the changes specifically attributable to placebo and nocebo mechanisms, including the neurobiological and psychological mechanisms of expectancies” (Evers et al., 2018). In this respect, this article will exclusively deal with the placebo and nocebo response in randomized controlled trials (RCTs), as little to nothing has been investigated related to the underlying mechanisms of the response in inflammatory bowel diseases (IBD), in sharp contrast to functional bowel disorders such as the irritable bowel syndrome (IBS) (Enck and Klosterhalfen, 2020), but also to other and specifically pain-associated disorders (Elsenbruch and Enck, 2015). However, as we have argued before (Enck and Klosterhalfen, 2019), meta-analyses and systematic reviews of RCTs in specific areas can generate substantial contributions to the understanding of the responses (Weimer et al., 2015a; Weimer et al., 2015b), even though they would need empirical, experimental validation. An excellent example for this is the old question of whether or not there are sex differences in the placebo response: As we (Enck and Klosterhalfen, 2019) have shown, experimental work underlines that men and women differ with respect to the utilized mechanisms (learning and expectation) for the placebo effects, while in clinical trials, these differences are equalized, resulting in similar placebo responses. A similar challenging task is evaluating whether or not age affects the placebo response and whether children exhibit more or less placebo/nocebo responses in RCT (Weimer et al., 2013; Weimer et al., 2015b)—this question still awaits its answer.

**FIGURE 1:**
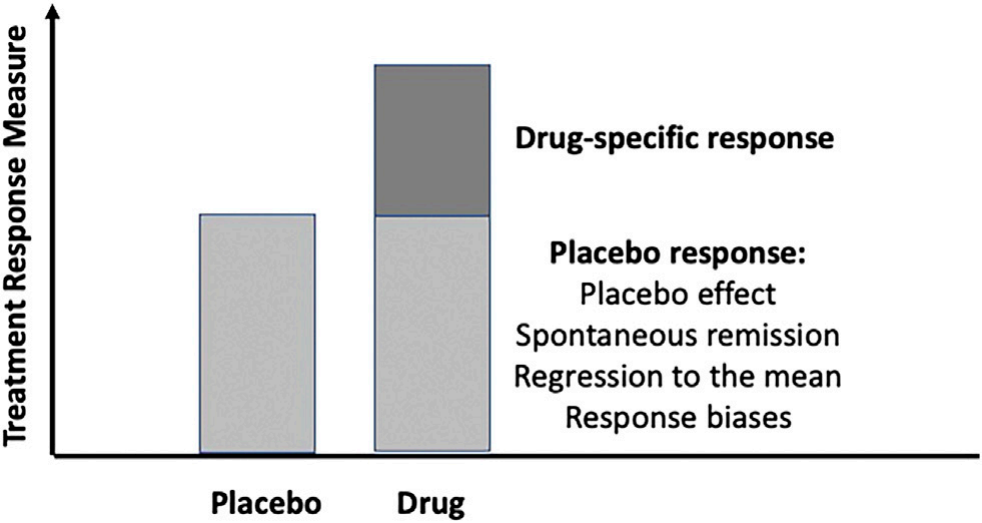
The conventional model of the placebo response in randomized, placebo-controlled trials (RCT): It is assumed that in the drug arm of the RCT, the placebo response is equal to its size in the placebo arm. In both, the effects of the spontaneous course of symptoms, methodological effects such as the “regression to the mean,” and responses biases contribute to its size, in addition to the placebo effect, which is the individual response of a patient towards placebo provision.

While we are aware that the placebo and nocebo responses have substantial effects on the design of clinical trials (Enck and Klosterhalfen, 2019), we will abstain from discussing this in more detail and rely on the traditional concept (Figure 1); that is, in placebo-controlled RCT, the placebo response includes not only the placebo effect (as defined above) but also other factors such as the spontaneous course of the disease if untreated and response biases and regression-to-the-mean effects. This is of specific relevance in chronic diseases where remission rather than healing is the primary goal of treatment. To uncover the contribution of spontaneous recovery, “no-treatment” control conditions would be needed that are deemed unethical in the care of IBD, at least in the acute disease condition.

Instead of questioning this principle concept, we imply that the global concept will remain valid, even if—for political, ethical, or other reasons—placebo-controlled trials are questioned or dismissed in the future. Comparator trials, where the placebo is replaced with an already drug available, have been shown to produce higher placebo response rates compared to placebos in a placebo-controlled trial; however, the true drug effect might be not identified and its efficacy tends to be overrated (Rutherford et al., 2009), and these trials require more patients to be included for statistical reasons (Weimer and Enck, 2014).

We will also abstain from discussing the option of “harnessing the placebo response” in clinical routine, beyond RCT (Enck et al., 2013), or implementing “open-label placebo” treatment (Ballou et al., 2017)—neither has yet been discussed for IBD patients. As mentioned above, IBD patients have rarely been exposed to placebo experiments, except in one brain imaging study (Schmid et al., 2015), discussed later.

In placebo research, nocebo responses and nocebo effects have only lately come into focus, which is evident from the small number of articles related to the topic in the specialized JIPS database (below): only about 650 of 4,500 genuine articles mention the term and a few have investigated such effects experimentally. Initially, the term was reserved for adverse events (AE) developed in RCT in the placebo arm of the trial, but the word “nocebo” has found a wider meaning, e.g., with the increased AE reports on patients switching from biologics to biosimilars in inflammatory diseases—we will get back to this later.

### Definitions, Mechanisms, and Measures

By definition (Evers et al., 2018), placebo responses refer to the (averaged) symptom response of a group of patients receiving a placebo during a randomized, placebo-controlled trial, e.g., of drugs. In contrast, the placebo effects refer to the individual change in symptoms that can be attributed to a known mechanism eliciting this change. Therefore, the placebo response in RCT includes factors that may not be driven by placebo effects but by the natural course of the disease or by methodological biases, e.g., repeated measures eliciting the “regression-to-the-mean” effect. The placebo effect in individual patients (as well as in many patients) is thought to be due to two major mechanisms: expectations that treatment will result in improved or disappearing symptoms because this is the reason for the treatment; learning, based on previous experience with the same or different symptoms, treatments, or medical settings. In experimental medicine, both factors can be separated to investigate their contribution, whereas in clinical medicine, they are usually may be compounded but their relative contribution may vary between individual patients. In theory, however, learning and expectations are not completely independent drivers of the placebo response. And finally, the nocebo responses or nocebo effects are thought to be governed by similar mechanisms (Elsenbruch and Enck, 2015; Enck and Klosterhalfen, 2019; Enck and Klosterhalfen, 2020)

## Methods

The origin of the Journal of Interdisciplinary Placebo Studies (JIPS) database has been described previously (Colloca et al., 2016; Enck, 2018). Instead of a complex search algorithm, the search term “placebo” is applied to the PubMed metadatabase, resulting in between 150 and 250 new citations per week that are screened for relevance for placebo research. Searches were and still are supplemented by hand-searched articles from other publications and meanwhile members of the scientific community that submit their newly published placebo articles. JIPS is also distributed to members of the Society of Interdisciplinary Placebo Studies (SIPS, www.placebosociety.com) for both service and supplementation. This database currently contains around 4,500 genuine placebo articles of all kinds, experimental data, reviews, and meta-analyses.

This database was started in 2004, and it has been searched retrospectively for all papers published until 2004 (approximately 100,000) and prospectively ever since. All identified articles are added to an EndNote-type database, including all metadata available in PubMed. This database (but not the PDFs collected) is accessible for the scientific community (upon registration on the JIPS website, www.JIPS.online). Since 2016, a monthly newsletter is distributed to subscribers that contains new citations for the IPS community and interested scientists.

The most recent use of the database was to conduct systematic reviews of specific areas (Colloca et al., 2016). We have employed it to identify knowledge gaps in placebo research, e.g., related to food and nutrition trials, sport interventions, and physical therapies and ethical, legal, and cultural aspects of the placebo phenomenon; similarly, we have determined that eHealth and mHealth applications of the placebo response/effects are clearly underrepresented, as compared to their current and future relevance (Enck et al., 2017). JIPS was screened for the terms “inflammatory bowel diseases” or “Crohns disease” or “colitis”, as of December 2020, for the purpose of this article.

## Results

The database contained (as of Dec 2020) nine meta-analyses of placebo-controlled RCT of Crohn’s disease (CD); however, there was substantial overlap between the meta-analyses. The largest of these (Jairath et al., 2017) reported data from 100 studies (depending on the read-out, see below), including 7,638 patients that received placebo, but excluded studies on CD patients postsurgery, which were exclusively covered by another meta-analysis (Renna et al., 2008) (15 studies). A more recent meta-analysis of five trials (Duijvestein et al., 2020) analyzed these trials after access to individual patient data rather than relying on published reports. Ford (Ford et al., 2014) finally added ten more studies specifically related to CD patients with fistulas, assessing partial or complete fistula closure. In the oldest meta-analysis, ten studies conducted before 1990 were covered (Salomon et al., 1992). In summary, therefore, there are data from 135 RCTs with approximately 10,000 CD patients.

The situation is similar in ulcerative colitis (UC). There are five rather large meta-analyses, with the latest and largest, with respect to the number of patients (Ma et al., 2018), including 51 RCTs with 5,182 individuals. Earlier RCT from an early meta-analysis (Ilnyckyj et al., 1997; Garud et al., 2008) and additional studies from more recent analyses (Jairath et al., 2016; Macaluso et al., 2019) included more than 150 RCTs with more than 10.000 UC patients. An early meta-analysis (but according to today’s standards, a systematic review) of 11 RCTs on treatment active colitis and five on patients in remission (Kornbluth et al., 1993) does not add much to the outcome, as it was not focusing on the placebo response but rather on the drug-placebo difference. To the best of our knowledge, only one meta-analysis has investigated placebo response rates in RCTs in patients with pouchitis (Athayde et al., 2018).

Depending on the clinical status of patients included in RCTs, different read-outs from studies need to be distinguished. In acutely diseased CD and UC patients, clinical improvement can either be partial, based on clinical, histologic, or endoscopic findings, or complete (achieving remission), based on similar criteria. In CD, improvement and remission were mostly based on the Crohn’s Disease Activity Index (CDAI). In patients in remission, in contrast, the efficacy of interventions is assessed either via maintenance of remission or via recurrence of symptoms, in both cases measured as the duration of being symptom-free.

One of the pitfalls of merging data from different meta-analyses is the fact that studies reporting more than just one drug arm, e.g., with different dosing or with different compounds, compared to one placebo arm, often are counted as two (or more) studies—but this is a questionable practice, since increasing the drug: placebo ratio also may affect the placebo (and drug) response rates (Papakostas and Fava, 2009). It is at least confusing when meta-analyses are performed with multiple endpoints, of which some are identified in all studies, whereas others are only found in some; hence, the numbers of studies and patients are inconsistently reported.

Finally, while most but not all studies attempted to identify single predictors of high or low placebo response but regression analysis, a few have gone beyond to construct multifactorial prediction models and one (Duijvestein et al., 2020) has done the latter based on individualized patient data rather than summary data from published articles, usually not available for drug studies.

### Size of the Placebo Response

Table 1 summarizes the pooled placebo response rates in CD and UC according to the meta-analyses performed and based on the clinical conditions and the respective endpoints of the studies.

**TABLE 1:** Pooled placebo responses (%) in randomized placebo-controlled trials according to different meta-analyses in Crohn´s Disease (CD), Ulcerative Colitis (UC) and pouchitis in patients with active disease or in remission (CDAI: Crohn´s Disease Activity Index).

Author	Year	No. of studies	No. of patients*	Active Disease		Patients in Remission		Not all studies have recorded the same outputs, hence the statistical basis for the pooled PE estimate may vary substantially
				Clinically Improved (%)	Achieving Remission (%)	Maintaining Remission (%)	Recurring of Symptoms (%)	
Crohn’s Disease (CD)								
Salomon	1992	10	339	19.6	28.4	37.5	–	different endpoints
Su	2004	21	707	19.0	18.0	–	–	CDAI defined
Gallahan	2010	20	1795	7–56	0–46	–	–	70/100 points CDAI decrease
Jairath	2017	100	7638	28.0	18.0	32	26	CDAI defined
Duijvesten	2019	5	580	16.2	5.2	–	–	>50% reduction in CD activity
CD Subgroups								
Pascua	2008	12	687	–	–	56	58	endoscopic evaluation
Renna	2008	16	799	–	–	–	23.7/50	relapse/severe relapse
Ford	2014	13	579	18.3	15.6	–	–	complete or partial closure
Ulcerative Colitis (UC)								
Kornbluth	1993	16	403	39.7	7.7/10.0	40.1	–	partial/complete/endoscopic
Ilnyckyj	1997	35	NR	26.7/30.3/25.2	9.1/13.5./8.6	–	–	clinical/endoscopic/histologic
Garud	2008	110	3982	32.1/40/36.6	23/28/–	53.1		clinical/endoscopic/histologic
Jairath	2016	51	4062	33	10/14	19	22	more interaction, duration
Ma	2018	64	5182	35	23/14	30	–	endoscopic/histologic
Macaluso	2018	31	2702	34/26	9	14	23/19	Mayo Score: clinical/histologic
UC-Pouchitis								
Athayde	2018	12	229	24	–	–	47	various pouchitis scores

As can be seen both across the meta-analyses and within some of them [e.g., (Gallahan et al., 2010)], the placebo response rates vary between 5 and 50% in CD and between 10 and 35% in UC, with respect to clinical improvement and remission. As expected, remission rates are lower than improvement rates, and the higher the standard of criteria (clinical vs. endoscopic), the lower the placebo response. Remission maintenance rates can also vary between 15 and 50%, following similar rules. Postsurgery placebo remission and recurrence rates are as high as 50%, while placebo response rates for fistula closures are lower, below 20%. Only one study has focused on pouchitis, but the reported 24% placebo clinical improvement response is well within the range of the effects in CD and UC.

Some of the variances between studies may be due to different sensitivities and specificities of the various IBD activity scores used or differences in the cut-off or response criteria, e.g., CDAI-based definition of remission. How much of these placebo responses can be attributed to a true placebo effect or to the natural course of the disease cannot be judged from these meta-analyses. We will discuss this further below.

### Predictors of the Placebo Response

While there is no consistent factor that has been reported in all or most meta-analyses, a few factors are listed more than once. They can be separated into disease, design, and other factors.

Disease factors: As with many other and noninflammatory diseases, lower disease activity at the beginning of the study was associated with higher placebo responses (Su et al., 2004; Jairath et al., 2016; Jairath et al., 2017); the same mechanism may be seen when “prior surgery, concomitant small bowel and colonic disease, fistulizing phenotype, or prior immunomodulator therapy” (Pascua et al., 2008), “prior exposure to TNF antagonists and increased concentrations of CRP at baseline” (Duijvestein et al., 2020), disease duration, and “ being non-naïve to anti-TNF’s"” (Macaluso et al., 2019) were found to be associated with lower placebo responses, while—not surprising—higher “placebo remission was associated with concomitant corticosteroids” (Ma et al., 2018; Macaluso et al., 2019).

**FIGURE 2:**
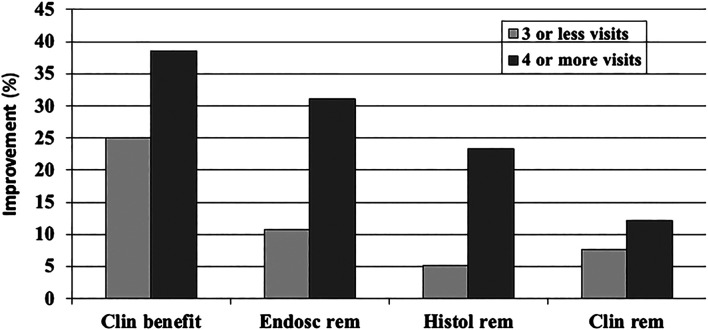
Dose-dependent function of the (pooled,%) placebo response in a meta-analysis of randomized and placebo-controlled trials (RCT) in ulcerative colitis (22): the number of doctor visits during the trial has an effect on all clinical outcome parameters, clinical benefit (improvement), endoscopically assessed remission, histologically assessed remission, and clinical remission measures.

Design factors: The study duration in CD (Su et al., 2004; Gallahan et al., 2010; Ford et al., 2014; Jairath et al., 2017) and UC (Garud et al., 2008; Jairath et al., 2016) is among the factors found to affect the placebo response, but data are less consistent in UC. Another factor associated with higher placebo responses is the number of study visits during the trials (Ilnyckyj et al., 1997; Su et al., 2004; Jairath et al., 2016) (Figure 2).

A factor that has frequently been discussed in areas outside IBD, e.g., in depression and other neurological and psychiatric diseases [e.g., (Enck, 2016)], was explicitly found not to be of relevance in IBD, i.e., the increase of placebo response rates in RCTs over time, with more recent studies exhibiting higher rates (Gallahan et al., 2010; Ma et al., 2018). The number of study centers was found to be effective as well, with more centers being associated with lower placebo responses (Jairath et al., 2017). One meta-analysis of UC trials found the country to determine the placebo response, with higher values in European countries (pooled improvement rate 36.4%) than those in the United States (24.9%) (Garud et al., 2008).

Other factors: It is not surprising that studies providing a new and expensive class of drugs such as biologics produce higher placebo response rates (Jairath et al., 2017). They also found the route of administration to be predictive of the placebo response—this is in line with data from the general literature: the more invasive procedures produce higher responses and injections are more effective than pills.

Increased placebo response can also be expected when instead of biomarkers and clinical disease signs, subjective, patient-reported outcomes (PRO) are used to assess the clinical efficacy of an intervention—this was shown in a meta-analysis of 16 RCTs in IBD (11 UC and 15 CD) in which IBS-specific (IBDQL) and generic (SF36) health-related quality of life was measured as the primary outcome (Estevinho et al., 2018): while overall drug-over-placebo benefit was maintained, the placebo response rates were high with 41% for clinical improvement and 31% for remission. This effect is as well established in the literature, but for many diseases, appropriate biomarkers are unfortunately missing. However, as has been shown recently that for IBD, fecal calprotectin may serve as such an appropriate biomarker (Bertani et al., 2020), possibly able to reduce (or limit) placebo responses, specifically if coupled with PRO. The important role of disease biomarkers is also evident in Figure 2 (above), where histological and endoscopic findings produce overall lower placebo response rates than clinical assessments, irrespective of the number of study visits.

### Placebo Effect Versus Spontaneous Remission (Natural Course)

To distinguish and parcel out true placebo effects from the placebo response as a summary of different mechanisms in RCT (Figure 1), it would be necessary to conduct a three-arm trial in which an equal fraction of patients would receive the active drug, the placebo, and no treatment, and this should be fully randomized and blinded—which of course is impossible for the “no-treatment” control, and ethically questionable as well. One way around this problem has been to install “waiting list controls” where patients are kept on a waiting list for a specific period of time before receiving treatment or being randomized into the RCT. In this case, the trial would be applied in clinical conditions of minor severity such as depression, nausea, and functional bowel disorders or with therapies other than drugs, e.g., with psychotherapy. Meta-analyses have shown that about 50% of the placebo response in RCT may be attributed to the spontaneous course of the disease—whether this holds true for IBD is an open question. When placebo arms of RCTs were used to estimate the natural course of IBD patients in remission (Meyers and Janowitz, 1989), about 50% of patients remained in remission for at least 1 year. Whether this allows concluding that a “true” placebo effect in IBS should be beyond 50% in remission trials remains to be known.

**FIGURE 3:**
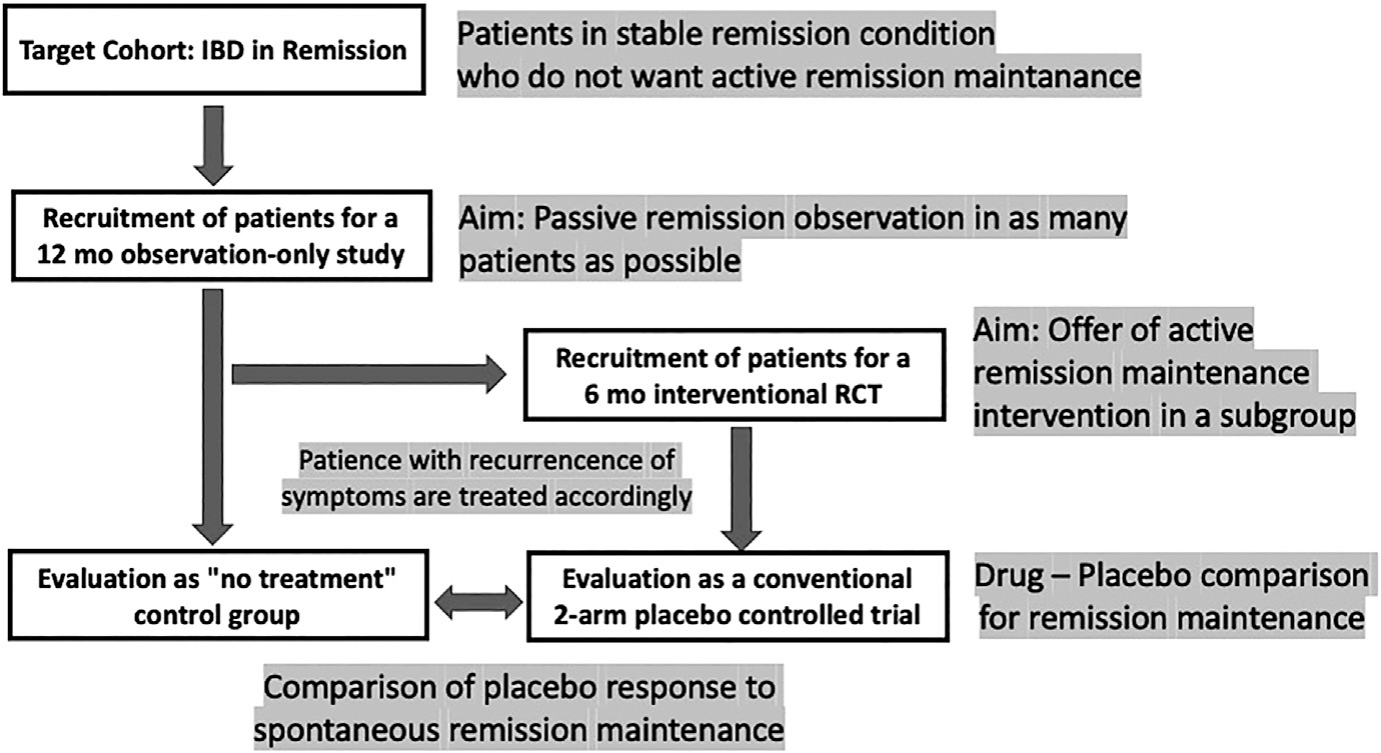
Design scheme for a remission maintenance study in IBD that would allow including a “no-treatment” arm to assess the spontaneous course of the disease. The concept has been called “cohort multiple randomized and placebo-controlled trials (cmRCT)” (Relton et al., 2010); for a discussion see (Weimer and Enck 2014).

However, there should be better ways to test this hypothesis, at least in maintenance trials, e.g., with the cohort multiple randomized controlled trial (cmRCT) (Relton et al., 2010) or the so-called Zelen design (Zelen, 1979). Such a trial would follow a stacked approach with double recruitment: first for an observation-only study, a large cohort of IBD patients in remission are recruited who are not taking any remission drug; subsequently, a subset of them is recruited for an interventional, placebo-controlled trial of a maintenance trial. This, to the best of our knowledge, has never been tried in IBD (Figure 3).

The ethical limitations of such a trial are at hand: leaving a group of patients with UC in remission untreated for some time can only be accomplished when narrow monitoring is warranted (Colombel et al., 2017), with patients who have minor symptoms during remission, and who, without the interference of the treating physician, would abstain from medication as long as possible anyway. Once remission is terminated, all patients need to receive appropriate standard treatment as soon as possible.

### IBD Versus IBS

During the discussion of the placebo responses in IBD, as above, it occurred to us that while the placebo response may be somewhat higher in IBS (across around 100 RCTs approximately 40% (Ford and Moayyedi, 2010)) than in IBD, the moderators and mediators were remarkably similar. As we have shown in recent systematic reviews (Enck and Klosterhalfen, 2020), these factors may be grouped in both conditions into disease factors (severity, duration, and pre- and concomitant treatments), design factors (duration of treatment, number of visits, number of centers, and number of study arms), individual conditions (age, sex, race, and proxies), and environmental factors (setting, therapists, healthcare plans, nationality, etc.). Clearly, many have been found to be in effect also in IBD, but whether they can be ruled out as relevant or irrelevant also depends on the focus these factors given in RCT and whether such determinants are part of the study reporting and publishing routine. These arguments have also been summarized by other studies (Sands, 2009). Probably the most neglected area in IBD research is the influence of the treating physician and his/her empathy and management skills in the therapy procedure—but that has probably surfaced in the IBS world only because of the lack of effective drug therapies.

To the best of our knowledge, only one study (Schmid et al., 2015) has compared central processing of placebo analgesia using an established experimental model in both patients with IBS and IBD (UC), compared to healthy control volunteers, and found that while IBS patients lacked downregulation of brain activity in relevant areas of the pain matrix during placebo application, no difference was observed in UC patients and healthy controls in terms of their ability of proper pain control, indicating normal placebo responses in UC.

### Nocebo Effects in IBD

To the best of our knowledge, there is only one meta-analysis (Ma et al., 2019) that has focused on AE reporting in the placebo arm of RCT, much in contrast to many such analyses in other areas of medicine, especially in the pain literature. This meta-analysis included 124 CD and 71 UC RCTs, so most of the trials that have also been included are the above placebo regression studies. The authors reported a pooled AE reporting of 70.6% in CD and 54.4% in UC and noted no differences in comparison to the respective active arms of the trials for AE reporting, severe AE reporting, and withdrawal due to AE, but a lower risk of symptom worsening in the active arms for both diseases. This is to some degree surprising given that overall AE reports are high compared to other reports on anti-inflammatory drug classes. This may support the notion that the high global symptom burden and the chronic nature of the IBD let the patients expect and tolerate substantially more AE anyway before leaving RCT.

Nocebo responses, the second aspect in IBD trials, are of a different nature, i.e., the report of increased AE when patients are switched from an established though expensive therapy with biologics to the more reasonably priced therapy with “biosimilars.” PubMed counts (as of Dec 2020) more than 50 publications in which biosimilar usage is linked to the term nocebo, despite the fact that direct (blinded) comparison of biologics with biosimilars usually does not result in higher AE and SAE reporting with biosimilars [e.g., (Boone et al., 2018)]. The nocebo reports in daily clinical routine, however, appears to be driven by media reports, self-aid groups, and Internet-based “fake” news. One may even wonder whether solely the selection of the term “biosimilar”—suggesting similarity but not equality—may have forced or supported this discussion.

However, since this phenomenon dominates medical practice but not medical research, e.g., in RCT, little is known about its mediators and moderators. It is, furthermore, not unique to biologics/biosimilars in IBD, but became visible also in other conditions, e.g., for statin use in blood pressure regulation (Pedro-Botet et al., 2019), lactose usage in individuals claiming lactose intolerance (Vernia et al., 2010), and wheat product consumption in nonceliac gluten hypersensitivity (Biesiekierski et al., 2013) and, in general, when patients are switched from a branded to a generic drug (MacKrill et al., 2019).

However, the pitfalls of this discussion are evident: with any drug taken, separating between an AE and a nocebo response is impossible in the individual patient, even if RCT provides some evidence for an AE being a nocebo response. Furthermore, nocebo responses are not personal characteristics of patients—anxiety may be a sign, but it is not proof of it. And as with the placebo response, the healthcare provider probably contributes as much or even more to a nocebo response as does the patient. In the early days of the biosimilar development, a survey among 1,200 physicians, nearly half of the physicians (and 43.8 in gastroenterology) questioned the equivalence and efficacy of biosimilars across many medical subspecialties (Cohen et al., 2017)—and this may reflect patients concerns and AE reports.

## Summary

While there are distinct differences between IBD and other conditions, especially IBS, the placebo response in RCT bears some similarities at different levels: the individual patient, the disease, the study design, and the healthcare system in general. Factors driving the placebo response have been identified in both conditions (IBD and IBS), but to a different degree. The size of the placebo response appears to be somewhat higher in IBS than in IBD, but in both cases, the contribution of the spontaneous course of the disease (e.g., spontaneous symptom recovery and remission) may contribute 50% or more to the placebo response. While nocebo responses in RCT are easy to identify—as AE in placebo arms, applying the label “nocebo effect” is difficult if at all possible in the individual patient in case of AE reports, e.g., following drug application or medication switch.

**TABLE 2:** Mediators and moderators of the placebo response in RCT in inflammatory bowel diseases (IBD) and the irritable bowel syndrome (IBS) and other functional gastrointestinal disorders, as evidenced (yes) in this and in previous reviews (e.g. Enck and Klosterhalfen 2020); question marks indicate a lack of knowledge.

IBD	Mediators/Moderators	IBS
Patient Factors		
?	age	yes
?	sex/gender	yes
?	personality	yes
?	race/culture	?
?	proxies	?
Disease Factors		
yes	severity of disease	yes
yes	duration of disease	yes
yes	previous treatments	yes
yes	concomintant therapies	?
yes	route of administration	?
Design Factors		
yes	No of study visits	yes
yes	No of study centers	yes
?	No of patients	yes
yes	Duration of RCT	yes
Therapist Factors		
?	Therapist (age, sex)	?
?	Clinical setting	yes
?	Health care system	?
yes	Nationality	yes
?	Culture	yes
